# 1034. COVID-19 Bloodstream Infection Outcomes Stratified by Social Determination Index (SDI) in NYC Public Hospital System

**DOI:** 10.1093/ofid/ofad500.065

**Published:** 2023-11-27

**Authors:** Chee Yao Lim, Kenneth Johan, Jorge Gutierrez, Alberto Romero Garcia, Afsheen Afzal, Mohannad Al-Khateeb Al-Khateeb, Boyana Yankulova, Mubarak Yusuf, Yinelka Silverio De Castro, Usha Venugopal, Addi Feinstein, Alexander La Fortune, Daniel Sittler, Vidya Menon

**Affiliations:** NYCHHC/Lincoln, New York, NY; NYCHHC/Lincoln, New York, NY; NYCHHC/Lincoln, New York, NY; NYCHHC/Lincoln, New York, NY; NYCHHC/Lincoln, New York, NY; Lincoln Medical Center, New York, New York; Lincoln Medical Center, New York, New York; Lincoln Medical Center, New York, New York; Lincoln Medical Center, New York, New York; Lincoln Medical Center, New York, New York; Lincoln Medical Center, New York, New York; NYCHHC/Lincoln, New York, NY; Lincoln Medical Center, New York, New York; Lincoln Medical Center, New York, New York

## Abstract

**Background:**

Impact of socioeconomic factors on outcomes was significant during the COVID-19 pandemic. We aim to study disparities in outcomes among hospitalized COVID-19 patients with bloodstream infection (BSI) stratified by SDI in the NYC Public Health System.

**Methods:**

Retrospective multicenter study of hospitalized patients with COVID-19 in 11 acute care hospitals in the NYC public health system from March 2020 to May 2020 was collected. Hospitals are further stratified according to the median household income of NYC using their zip code area into high (≥$65,000), medium ($40,000-64,999) and low income (< $40,000). Descriptive statistics were used to compare baseline patient characteristics including COVID-19 severity and BSI features. The primary outcome was in-hospital mortality. Logistic regression models were used to adjust for potential confounders.

Inclusion Process of Study Population

**Figure:**
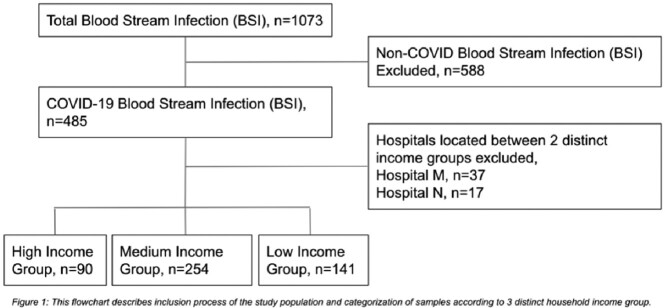

**Results:**

Eleven hospitals were stratified based on SDI (high income: 2, medium income: 4, low income: 3), 2 were excluded as they lie on zip codes with overlapping income. Patients’ baseline and BSI characteristics are described in Table 1. For every change in income group: high-middle and middle-low, the odds of mortality increased by 1.33 times (p=0.048). Additionally, for every increase in 1 year of age above 23, the risk of mortality from BSI increased by 2.2% (p=0.004).

**Figure:**
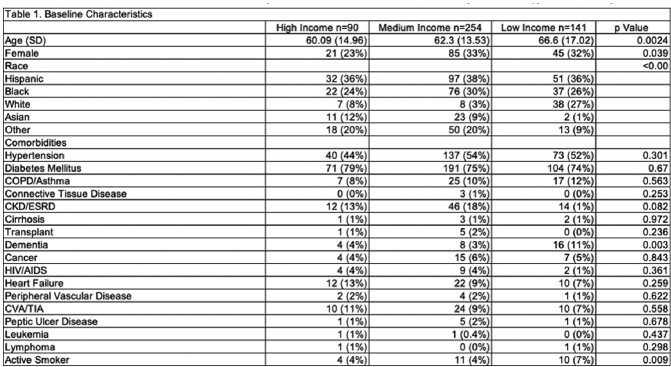

Baseline Characteristics of Subjects Stratified by Household Income Status

**Figure:**
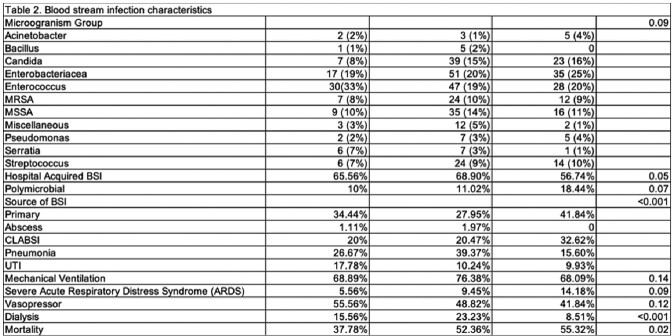

Bloodstream Infection Characteristics Stratified by Household Income Status

**Conclusion:**

The study shows the impact of health disparities during COVID-19 among hospitals with the same standard of care, where higher mortality rates were observed in low and middle-income zip code areas in NYC, likely due to the burden of social determinants of health. Hospital acquired BSI was uniformly high in all income groups, compared to primary BSI. Nonetheless, it provided an insight about the need for further investigation about the impact of socioeconomic factors on mortality and for strategies to address disparities in access to healthcare.

**Disclosures:**

**All Authors**: No reported disclosures

